# Improvement of primary care for patients with chronic heart failure: A study protocol for a cluster randomised trial comparing two strategies

**DOI:** 10.1186/1748-5908-6-28

**Published:** 2011-03-25

**Authors:** Jan van Lieshout, Betty Steenkamer, Marjan Knippenberg, Michel Wensing

**Affiliations:** 1 Scientific Institute for Quality of Healthcare, Radboud University Nijmegen Medical Centre, P.O. Box 9101, 114 IQ Healthcare, 6500 HB Nijmegen, The Netherlands; 2 Robuust, Le Sage ten Broeklaan 11, 5615 CP Eindhoven, The Netherlands

## Abstract

**Background:**

Many patients with chronic heart failure (CHF), a common condition with high morbidity and mortality rates, receive treatment in primary care. To improve the management of CHF in primary care, we developed an implementation programme comprised of educational and organisational components, with support by a practice visitor and focus both on drug treatment and lifestyle advice, and on organisation of care within the practice and collaboration with other healthcare providers. Tailoring has been shown to improve the success of implementation programmes, but little is known about what would be best methods for tailoring, specifically with respect to CHF in primary care.

**Methods/design:**

We describe the study protocol of a cluster randomised controlled trial to examine the effectiveness of tailoring a CHF implementation programme to general practices compared to a standardised way of delivering a programme. The study population will consist of 60 general practitioners (GPs) and the CHF patients they include. GPs are randomised in blocks of four, stratified according to practice size. With a tailored implementation programme GPs prioritise the issues that will form the bases of the support for the practice visits. These may comprise several issues, both educational and organizational.

The primary outcome measures are patient's experience of receiving structured primary care for CHF (PACIC, a questionnaire related to the Chronic Care Model), patients' health-related utilities (EQ-5D), and drugs prescriptions using the guideline adherence index. Patients being clustered in practices, multilevel regression analyses will be used to explore the effect of practice size and type of intervention programme. In addition we will examine both changes within groups and differences at follow-up between groups with respect to drug dosages and advice on lifestyle issues. Furthermore, in interviews the feasibility of the programme and goal attainment, organisational changes in CHF care, and formalised cooperation with other disciplines will be assessed.

**Discussion:**

In the tailoring of the programme we will present the GPs a list with barriers; GPs will assess relevance and possibility to solve these barriers. The list is rigorously developed and tested in various projects. The factors for ordering the barriers are related to the innovation, the healthcare professional, the patient, and the context.

CHF patients do not form a homogeneous group. Subgroup analyses will be performed based on the distinction between systolic CHF and CHF with preserved left ventricular function (diastolic CHF).

**Trial registration:**

ISRCTN: ISRCTN18812755

## Background

Chronic heart failure (CHF) is a highly prevalent chronic disease with high morbidity and mortality rates. The prevalence of CHF in the Western world is 1% to 2% in the general population and at least 10% among the age group of 85 years and older [1,2]. Mortality in CHF patients is high compared to their age group [2,3]. High morbidity is associated with high hospital admission rates and reduced quality of life [4]. There are effective, evidence-based treatments which improve mortality and morbidity but use of and adherence of treatments is suboptimal despite clinical guidelines [5-9].

A large group of patients with CHF receive treatment in primary care. There are many programmes for the management of long-term conditions in primary care -- *e.g*., diabetes, COPD and depression -- but at present not for CHF, though several disease management programmes exist for CHF related to outpatient clinics. As a substantial proportion of CHF patients do not attend such clinics but visit their GP instead, these patients are not enrolled in a structured care programme.

To improve the management of CHF in primary care, we have developed an implementation programme, comprising both educational and organisational components, the latter aimed at improved and structured care. We pilot tested this programme in 19 general practices [10]. The pilot programme was targeted at implementing the prevailing practice guideline for general practice [11]. In the mean time, in the Netherlands an interdisciplinary guideline on CHF, based on the European Society of Cardiology (ESC) guideline, was developed and published in May 2010 [12]. So, we adapted the implementation programme according to this new guideline, taking into account the lessons learned in the pilot study.

Apart from offering a standard implementation programme, another approach is to tailor an intervention to the special needs and conditions in a general practice. Ideally, tailoring has three components: identification of factors associated with implementation (or labelled as 'barriers to change'), to match implementation interventions to those factors, and evaluation of the tailored implementation programme. Tailoring has been shown to have a modest effect on the success of the implementation programme [13], but little is known about what would be best methods for tailoring [14]. In addition, research evidence specifically supporting this hypothesis with respect to improving primary care for CHF patients is not yet available. In one study no relation was found between barriers perceived and ACE inhibitor prescription behaviour [15]. Earlier studies to improve CHF patients care focused on disease management programmes, mainly case management, and on education and drug prescription [16,17].

Patients being clustered in practices, we planned to conduct a cluster randomised controlled trial with randomisation at the practice level. The aim of the study will be to examine the effectiveness of tailoring the implementation programme to general practices compared to a standardised way of delivering the implementation programme to general practices.

## Methods

The project will be a collaborative project of IQ healthcare, a research department of Radboud University Nijmegen Medical Centre, and the four regional supportive structures for primary care ('ROS') in the south of the Netherlands and their cooperative bond Robuust. The medical ethical committee (CMO Regio Arnhem -- Nijmegen) assessed the study proposal and waived approval.

### Participants

The study population will consist of 60 general practitioners (GPs) and the CHF patients they will include. GPs will be recruited in the south of the Netherlands, contacted either directly or indirectly via various regional organisations by advisors of the regional supportive structures for primary care. GPs will be informed about the project, and when they agree to participate they will be instructed to send in an admission form with data on practice organization necessary for stratification and randomization.

Evidence of change will be assessed by studying the effects of the intervention on patients with CHF registered with the participating GPs. As in the pilot [10], GPs will include all CHF patients from their practices who are over 18 years of age of whom the GPs consider themselves to be the physician taking care of the treatment of this condition in the patient. We expect eight to ten patients per practice [18,19]. Patients will be sent explanation about the study and asked for informed consent to participate in a patient questionnaire. Data collection will be anonymized.

## Interventions

### Standardised delivery of the implementation programme

The implementation pack contains educational materials for the professionals and patients. There is a recommended protocol for multidisciplinary management and a template for clinical care presented as a guiding registration form. Furthermore, we offer support by a visiting practice consultant and the possibility to contact a GP with a special interest in CHF management.

The project materials from the pilot were amended following the pilot study and the development of the new interdisciplinary practice guideline [12]. Participating GPs will be informed about healthcare professionals from other primary care disciplines, *e.g*., dieticians and physiotherapists, and these professionals will be informed about the project, encouraging the role of the multidisciplinary team in CHF management. The paragraph on non-pharmaceutical treatment was enlarged and the pharmaceutical paragraph had some changes. Now, recommendations on drug treatment are different for patients with systolic CHF compared to patients with a preserved systolic heart function, so called diastolic CHF (See Table 1). Finally, new recommendations on the use of devices were formulated.

**Table 1:** Changes in medication advice

Medication advice based on the 2005 GP practice guideline	Medication advice based on the 2010 multidisciplinary guideline
One scheme:	Systolic heart failure:
- Diuretics	- ACE inhibitor (or ARB if not tolerated) in evidence based doses
- ACE inhibitors or ARB	- Diuretics for fluid retention
- Beta blocker	- Beta blocker blockers licensed for heart failure in evidence based doses
- Aldosteron antagonist	- Aldosterone antagonist or ARB
- Digoxin	- Digoxin or H + ISND
	
	Diastolic heart failure:
	- Diuretic if signs of fluid retention
	- Adequate treatment of co morbidity
	- Strict blood pressure control

ARB = angiotensin II receptor blocker

H + ISDN = Hydralazine + isosorbide dinitrate

The paper template is used to direct care and collect data to demonstrate the effectiveness of the interventions. Demographic data is collected in particular the aetiology of the CHF and the diagnostic category, whether diagnosis was echocardiography based, on the existence of diastolic or systolic CHF, and pharmaceutical treatment at the start of the project period. A second page poses questions about non-pharmaceutical issues such as advice about physical activity and influenza vaccination. Finally, we offer different forms for patients with systolic or diastolic CHF, based on the recommendations on medication as summarised in Table 1.

The pilot study demonstrated that three practice visits was the optimum number and these shall be offered to all participating practices. The practice visitor is an educational facilitator trained in supporting behaviour change in practices.

The pilot study also demonstrated that there was little multidisciplinary clinical activity in the improvement of care for CHF patients, and this has been addressed in this project. We now use the multidisciplinary practice guideline as a starting point instead of the monodisciplinary GP's practice guideline. Furthermore, the regional advisors will determine the social network in the practice area, providing information on other primary care disciplines, *e.g*., dieticians and physiotherapists, with extra expertise and interest in CHF treatment. These workers in the other disciplines will be informed about the project and receive relevant information in line with the multidisciplinary guideline.

All materials are offered paper based in a binder. We will also present all materials on a website and examine the possibilities for designing the guiding patient registration forms on this website as well.

### Tailored delivery of the implementation intervention

The intervention group of practices will have the agenda of their practice visits determined by the results of a questionnaire identifying the barriers they perceive to the introduction of a programme for the management of CHF in primary care. The barriers listed in the questionnaire are based on previous research and grouped in relation to the innovation, the healthcare professional, the patient, and the context [20]. GPs are asked to indicate the relevance of each barrier in their practice situation on a five-point scale, and whether or not they think that the barrier can be solved. Table 2 shows some examples of barriers suggested in the format of the questionnaire. GPs are offered the possibility to add barriers they perceive that are not yet identified. When barriers are identified, those that are relevant and solvable will be prioritised and addressed during the practice visits.

**Table 2:** Examples of possible barriers as presented in the GP's questionnaire for the tailored intervention.

		To what extend is this barrier relevant for your practice situation?	Do you consider it possible to solve this barrier in your practice situation?
1.	**Innovation**		

	The recommendations in the multidisciplinary practice guideline heart failure ask for changes in our existing practice routines that are too big.	❑ Not at all ❑ A little ❑ Neutral ❑ Relevant ❑ Very relevant	❑ Yes ❑ No ❑ Doubtful

2.	**Health care professional**		

	I lack sufficient knowledge of the recommendations in the multidisciplinary practice guideline heart failure.	❑ Not at all ❑ A little ❑ Neutral ❑ Relevant ❑ Very relevant	❑ Yes ❑ No ❑ Doubtful

3.	**Patient**		

	Considering patients with complex health issues all time reserved usually is taken completely by the actual problems and we do not manage to provide a more structured approach for the heart failure problems.	❑ Not at all ❑ A little ❑ Neutral ❑ Relevant ❑ Very relevant	❑ Yes ❑ No ❑ Doubtful

4.	**Context**		

	We lack sufficient supportive staff to provide care according to the practice guideline.	❑ Not at all ❑ A little ❑ Neutral ❑ Relevant ❑ Very relevant	❑ Yes ❑ No ❑ Doubtful

### Objectives

The objective of the study is to examine the effectiveness of identifying barriers to change, and tailoring education and support in comparison with a standard intervention programme, to improve the management of patients with CHFin primary care.

Our null hypothesis is that tailoring does not result in better implementation of the guidelines for CHF compared to a standardised delivery of our implementation programme. In addition, we will also examine whether either of the two programmes are associated with improvements in healthcare delivery and patient outcomes.

### Measures

Data collection will comprise the following measures: patient questionnaires, patient registration forms, and telephone interviews with the GPs. Practices will send out patient questionnaires based on validated and previous used questionnaires: PACIC [21], EQ-5D [22], questions about 'continuity of care,' and Morisky's questionnaire on medication adherence [23]. In the patient registration forms, data about non-pharmaceutical and drug therapy are registered by the GPs and their staff: they register the baseline treatment at inclusion, and during the patient contacts throughout the study period they register all advice given, referrals, drug therapy changes, and information on hospitalisation and mortality. The GP interview will score goal attainment and a qualitative assessment of the programme. All measures have been applied by our group in previous studies.

### Outcomes

The primary outcome measures are determined at the patients' level and consist of experience of receiving structured primary care for CHF (PACIC) and health-related utilities (EQ-5D), and of drug prescriptions.

PACIC is a questionnaire consisting of 27 items, which is related to the Chronic Care Model [24]. We translated and validated PACIC for use in general practice in the Netherlands [25]. EQ-5D consists of five questions and a Visual Analogue Scale (VAS), which has been validated for use in the Netherlands [26]. These outcome measures reflect key aspects of medical treatment, organization of care, and patient reported quality of life, and were chosen pragmatically because of the availability of relevant, validated, responsive, composite measures.

Drug prescription scores are assessed using the guideline adherence index (GAI), indicating the proportion of indicated drugs that is prescribed [27,28]. These include ACE inhibitors and beta blockers, and -- depending on the patient's CHF severity according to the NYHA classes -- further drug classes [12].

Secondary outcome measures at the patients' level concern lifestyle advice and medication during the study period, based on the patient registration forms providing information on non-pharmaceutical advice and drug prescriptions, continuity of care, and medication adherence. Considering drug therapy we will assess the percentages of ACE inhibitors and beta blockers prescribed in the evidence-based target dosages. Secondary outcome measures at the practice level are goal attainment and qualitative assessment of the programme, focussing on the subjective GP's experiences, for instance on improvement of practice organisation and collaboration with other primary care professionals.

### Randomisation

The study will be a randomised controlled trial, with a one-year follow-up period. Practices are stratified according to practice size as solo, duo, or group practice. On inclusion, practices are assigned to one of the study groups using randomisation in blocks of four per stratum by a research assistant. Blocks were generated electronically with the help of a statistician. The randomising procedure itself is concealed for practices, consultants, and researchers (JvL, MW). The regional advisors including the practices have no access to the randomisation process. After randomisation, both the practice and the regional advisor are informed. The intervention is open to all involved.

### Data analysis

The primary analysis is a comparison of primary outcomes at follow-up between the study groups, taking into account clustering of patients within practices. Multilevel regression analyses will be used to explore the effect of practice size and type of intervention programme. In addition, we will examine both changes within groups and differences at follow-up between groups with respect to percentage of patients on the maximum tolerated dosage of ACE inhibitor or angiotensin II receptor blocker and of beta blockage; medication completely according to the guideline; having received influenza vaccination in the winter season 2010-2011; having received professionally guided physical activity training; and the number of non-pharmaceutical issues addressed. If possible, subgroup analyses will comprise type of CHF (systolic or diastolic) and diagnostic certainty (secondary care diagnosis, echocardiography diagnosis) The interviews will be used to assess the feasibility of the programme and goal attainment, organisational changes in CHF care, and formalised cooperation with other primary care disciplines and specialist care.

### Sample size

Power calculations are based on the primary outcome measurement PACIC. The PACIC comprises 27 questions with an answering scale from 1 to 5. A previous unreported study showed that its standard deviation was 1.0. To detect a difference of 0.3 points with a standard deviation of 1 (a relatively small effect), with alpha = 0.05 and beta = 0.20, ICC = 0.05, a total of 51 practices with 10 patients each would be required, or 59 practices with 8 patients.

### Time frame

Practice inclusion is planned from June until October 2010. After inclusion, practices participate in the programme during one year. At the start of the year, the GPs include all their patients with CHF they treat at least in part themselves. During that year, patients with newly diagnosed CHF can also be included. Data management in the patient registration forms is a continuous process during the project year. After the project period of a year, the forms are anonymised and sent to the researchers coded. Furthermore, practices will send coded patient questionnaires with explanation of the survey and ask for an informed consent; these will be returned to the researchers. Finally the researchers will contact the GPs participating for the telephone interview. The GPs will receive feedback based on the patient registration forms and the questionnaires.

## Discussion

There exist various classifications of barriers (and facilitators) and multiple approaches for linking interventions to barriers [29-31]. In their systematic review, Légaré and colleagues propose a conceptual framework with knowledge, attitude, and behaviour as main factors [31]. Our choice of barriers is based on a list rigorously developed in the Netherlands with a literature study in 2002, expert input, and tested in various projects in the Netherlands [20]. Our main factors for ordering the barriers are related to the innovation, the healthcare professional, the patient, and the context. The barriers presented in this study correspond very well to the barriers found most often in the Légaré review, including time pressure and lack of applicability. Looking for the barriers for each individual practice and not for the practices participating in general, we decided to have the barriers proposed scored in email contact with the GPs in two rounds.

Several studies were conducted to assess the effect of tailoring implementation. In a systematic review, the authors conclude that identifying barriers and tailoring intervention to these barriers may improve clinical practice compared to no intervention and to guideline dissemination. In their meta analysis including eight studies identified with a non-tailored intervention in the control group, they report a modest effect of tailoring [13].

All data collection will take place at the end of the project period. Within the context of the evaluation of the implementation project, it is not feasible to have data collection both at the start and at the end, but the patient registration form will have baseline data on treatment issues. This also will allow for within group analyses, giving information about the effectiveness of both implementation strategies separately. Unfortunately, this data collection, either paper based or electronically, will require additional administrative actions from the practices. Enabling data collection from the electronic medical record will be beyond the scope of this project. Considering the patient questionnaire's results, with one measurement at the end, this restricts us to intergroup analyses.

Our sample size is based on power calculation considering the PACIC outcomes. In previous research, a difference of 0.5 was found in the evaluation of a CHF and a diabetes programme [32,33]. Thus, an effect size of 0.3 on PACIC can be considered a modest effect. We aim at a lowered effect because we compare two strategies that are both to be expected to have some impact.

Drug prescription is also a primary outcome measure. We will present the GAI as a measure for the percentage of indicated drugs prescribed. In previous research, many patients appeared to receive suboptimal dosages. When considering ACE inhibitors and beta blockers, we will report on the percentage of prescriptions in the indicated, high-target dosages [34].

We decided to stratify practices included based on practice size. There is some evidence of a relation between practice size and quality of care [35,37]. In one study, larger practice size was associated with more structured care [35]. Stratification of practices based on practice size could be defined as the number of GPs in the practice and based on patient list size. We choose a stratification scheme based on the former, with the strata solo, duo, and group practices, which appeared feasible in previous research leading to strata of comparable size.

CHF patients do not form a homogeneous group; apart from aetiology, we make a distinction between systolic CHF and CHF with preserved left ventricular function (diastolic CHF). In our study, both patient groups may be included, as the practice guideline gives recommendations for both the patients with and without left ventricular systolic dysfunction. Subgroup analyses will be performed.

## Competing interests

Michel Wensing is an Associate Editor of Implementation Science. All decisions on this manuscript were made by another senior Editor. The authors declare that they have no competing interests.

## Authors' contributions

JvL and MW designed the study; JvL drafted the materials concerning the content; BS and MK made essential contributions to the design and the devolvement of the materials and the practical implications. JvL drafted and revised the manuscript; MW, BS, and MK commented it critically. All authors approved the final version of the manuscript.
